# Job boredom as an antecedent of four states of mental health: life satisfaction, positive functioning, anxiety, and depression symptoms among young employees – a latent change score approach

**DOI:** 10.1186/s12889-024-18430-z

**Published:** 2024-03-27

**Authors:** Jie Li, Janne Kaltiainen, Jari J. Hakanen

**Affiliations:** 1 Faculty of Social Sciences, Tampere University, Tampere, Finland; 2 Finnish Institute of Occupational Health, Work Ability and Working Careers, Helsinki, Finland

**Keywords:** Job Boredom, Life Satisfaction, Positive Mental Health, Depression, Anxiety, Latent Change Score Modelling

## Abstract

**Background:**

Job boredom has been generally associated with poorer self-rated health but the evidence is mainly cross-sectional and there is a lack of a holistic mental health approach. We examined the temporal relationships between job boredom and mental health indicators of life satisfaction, positive functioning, anxiety, and depression symptoms.

**Methods:**

We analyzed a two-wave postal survey data of adults aged 23 to 34 that was collected from the Finnish working population between 2021 and 2022 (*n* = 513). Latent change score modelling was used to estimate the effects of prior levels of job boredom on subsequent changes in mental health indicators, and of prior levels of mental health indicators on subsequent changes in job boredom.

**Results:**

Job boredom was associated with subsequent decreases in life satisfaction and positive functioning and increases in anxiety and depression symptoms. Of these associations, job boredom was more strongly associated with changes in positive functioning and anxiety symptoms than with changes in life satisfaction.

**Conclusions:**

Our two-wave study suggests that job boredom, a motivational state of ill-being in the work domain, spills over into general mental health by decreasing life satisfaction and positive functioning and increasing anxiety and depression symptoms. Our findings contribute to the understanding of the potential detrimental effects of job boredom and its nomological network. From a practical perspective, workplaces are adviced to improve working conditions that mitigate job boredom and thus promote employees’ mental health.

**Supplementary Information:**

The online version contains supplementary material available at 10.1186/s12889-024-18430-z.

## Background

Previous studies have indicated that job boredom is prevalent in various occupations [1, 2] and especially among younger workers [1, 3]. While studies examining job boredom are increasing, there is a lack of evidence on its temporal associations with multifaceted mental health indicators. Concerns about mental health are increasing globally [4]. For instance, a recent report by the Social Insurance Institution of Finland shows that mental health-related issues are the most common reason for applying for sickness benefits among under 35-year-old adults [5]. Furthermore, the COVID-19 pandemic may have further challenged workers' well-being, as increases in teleworking have potentially increased job boredom [3]. For employees, employers, and societies, establishing practices that foster well-being at work is critical as past research has shown spillover effects from work-related well-being to general mental health indicators [6] and vice versa [7]. Yet, most studies have adopted a pathological perspective, focusing only on mental illness symptoms, which does not consider the holistic nature of mental health, that is, also its positive dimensions.

Keyes [8, 9] argues that positive and negative mental health symptoms exist in separate continuums. Therefore, only the absence of negative mental health symptoms may not be a sufficient condition for having “good” mental health as also presence of positive mental health is desirable. From the perspective of this dual-continua model of mental health [10], it is essential to examine mental health as a holistic concept as job boredom may have different associations with different mental health indicators.

Depression and anxiety symptoms have long been popular indicators of mental health. While they are both indicators of mental illness, they have distinct symptomatology. Anxiety is commonly defined by symptoms such as nervousness and trouble relaxing [11] whereas depression is defined by loss of enjoyment in life [12]. From the perspective of positive mental health, life satisfaction has typically been the most studied indicator. While life satisfaction is an important aspect of positive mental health, it mainly refers only to positive affect [13]. From a more comprehensive perspective, positive functioning represents the more psychological aspect of positive mental health [8]. Positive functioning refers to overall positive socio-psychological functioning, such as purpose in life, feeling competent, being engaged in daily activities, and actively contributing to the relationships with others [14]. Following Keyes [8, 9], we adopt a holistic model of mental health by examining two indicators of positive mental health (life satisfaction and positive functioning) and two indicators of negative mental health (anxiety and depression symptoms).

Previous cross-sectional research has found job boredom to be associated with various negative work-related outcomes such as turnover intentions [1], job dissatisfaction [15], and low organizational commitment [16]. Recent research also suggests that job boredom may be detrimental to one’s health [17]. These findings provide initial evidence of the importance of mitigating job boredom from the organizations’ perspective. Regarding mental health, previous cross-sectional studies suggest that job boredom is associated with depression symptoms [18, 19], anxiety symptoms [20], lower positive mental health [21], and general dysphoria [22]. While studies on the daily experience of job boredom have found it to be associated with a more depressed mood later in the evening [23], there still is a lack of knowledge about the potential long-term effects of job boredom on mental health.

Taken together, there is a clear lack of evidence on whether job boredom is more likely an antecedent of mental health or vice versa, and more fundamentally, whether these experiences are temporarily related over the longer term. Moreover, previous studies have focused on illness symptoms, thus sidelining indicators of positive mental health. This gap limits our understanding of the potential effects that job boredom and mental health may have on each other and thus limits practitioners from establishing new and effective approaches to promote general mental health and mitigate boredom at work.

We draw from the conservation of resources theory (COR) [24, 25] to examine the dynamic spill-over effects between job boredom and mental health indicators. According to the principles of COR theory, resources in general are aspects that individuals perceive as valuable. Furthermore, resources do not exist separately but rather they are reciprocally related. Those with greater resources are more capable of gaining more resources and less vulnerable to resource loss. Conversely, those with fewer resources, are more prone to further resource loss and less capable of resource gain.

In this study, we operationalize job boredom, anxiety, and depression symptoms to represent lack of resources. Life satisfaction and positive functioning in turn represent the presence of resources. Job boredom indicates low employee well-being and motivation [26] and thus, predisposes individuals to suffer subsequent resource losses which we expect to be manifested as increases in anxiety and depression symptoms. Furthermore, bored employees are less capable of resource gain which would manifest as decreases in life satisfaction and positive functioning. Conversely, life satisfaction and positive functioning protect against further losses, which would manifest as decreases in job boredom. Similarly, anxiety and depression symptoms are expected to lead to increases in job boredom as it indicates a resource loss.

While we expect job boredom to be associated with all four mental health indicators, the strengths of these associations might differ. For example, following the rationale of the dual-continua model [10], for instance, a given state or experience may increase negative mental health while not having the same effect on decreasing positive mental health. Job boredom is a unique type of employee ill-being as it is characterized by low stimulation rather than overstimulation [27]. Furthermore, being anxious is characterized as a high arousal state [28], similar to positive functioning [13]. Therefore, we expect job boredom to be more strongly associated with anxiety symptoms and positive functioning as opposed to life satisfaction and depression symptoms, which are fundamentally characterized as affective states [13, 28].

Taken together, we contribute to the question if job boredom is an antecedent of general mental health, examined as a multifaceted phenomena, and/or vice versa. We also examine if some associations are stronger compared to others and provide further understanding regarding the potential losses and gains as we estimate changes in outcome variables.

## Methods

### Participants and procedure

We collected two-wave survey data from the Finnish population of adults between the ages of 23 to 34. The selected age group was deemed to roughly reflect young adults in the Finnish context. Contact details of 12 000 individuals aged 23 to 34 were drawn randomly from the Finnish population registry in early 2021. The baseline survey (T1) was sent to these individuals via mail during mid-May 2021 and the follow-up (T2) was sent in late April 2022. At both times, data were collected for roughly one and a half months including two reminder letters. At T1, 1794 participants (15%) returned completed surveys of which 1628 agreed to be contacted for a follow-up. At T2, 738 participants (45%) completed the follow-up survey. For the present study, we included those participants who worked (full-time, part-time, or occasionally) during both waves and reported at least 10 or more weekly working hours during both waves. Altogether, 516 participants were included in the analysis. Analyses of the possible impact of non-response bias are presented in the Supplementary materials. Overall, differences between respondents and non-respondents were small and we do not expect them to bias our findings. We also weighted the analyses in terms of gender and age to correspond to 2021 population characteristics.

### Variables

All the item wordings and scales are presented in the Supplementary material. Job boredom was measured by drawing three items from the Dutch Boredom Scale [16]. These items represented the behavioural, cognitive, and affective aspects of job boredom. Life satisfaction was measured by asking ‘*Overall, how satisfied are you with your life?*’. Positive functioning was measured using the Flourishing Scale [14] which has eight items that capture aspects of positive socio-psychological functioning, such as contributing to relationships and engaging in daily activities. Anxiety symptoms were measured using the Generalized Anxiety Disorder measure [11] which has seven items that capture typical anxiety disorders such as anxiousness, excessive worrying, and trouble relaxing. Depression symptoms were measured using six items drawn from the Four-Dimensional Symptom Questionnaire [12] that represent loss of meaning, joy, and overall helplessness. Age, gender, education, job transition, and telework were used as control variables. Education was dichotomized as having either a university or higher degree and other post-secondary degrees or lower. Because teleworking has become increasingly common after the COVID-19 pandemic and may impact job boredom [3, 29] we included it as a control variable. Teleworking was measured by asking ‘*How often, on average, have you worked remotely in the last six months?*’. The response scale was from 1 ‘*not at all*’ to 5 ‘*approximately all the time*’.

### Analysis

The analysis was conducted using Mplus v.8 [30] and maximum likelihood estimation with robust standard errors to account for the non-normal distributions of anxiety symptoms (T1 skewness: 1.34, T1 kurtosis: 1.78; T2 skewness: 1.46, T2 kurtosis: 1.96) and depression symptoms (T1 skewness: 3.65, T1 kurtosis: 16.32; T2 skewness 3.88, T2 kurtosis: 17.26). Due to the conceptual overlap between the anxiety and depression symptoms, the items for anxiety and depression measures were parcelled so that they could be more clearly distinguished from each other, as recommended by Little et al. [31]. Strong measurement invariance was supported before the main analysis, suggesting that our factor structure for latent variables were consistent over time in terms of similar item loadings and intercepts. For detailed information about the parcelling and measurement invariance process, see the Supplementary material.

To examine the associations between job boredom and mental health indicators, we used latent change score modelling (LCSM). Latent change scores capture intraindividual changes in latent constructs and were used as a technique to model the outcome variables in our study. By this, the model showed whether the T1 predictor was associated with the T1-T2 change in the outcome variables. We estimated the latent change scores (ΔT1-T2) by fixing the path coefficients to 1 from the T1 and the ΔT1-T2 variables to the T2 variable. Then, we regressed the T1 variable to the ΔT1-T2 to account for the autoregressive path and set the T2 variance to 0 as it was defined by the T1 and ΔT1-T2 variables. The remaining ΔT1-T2 variable represented the within-person change in the latent construct, and for the variables that we measured with multiple items (all others except life satisfaction), these constructs were also free of measurement error [32, 33].

We estimated four models to determine the best-fitting model for our data. First, we estimated the autoregressive model (Model 1), in which all T1 variables predicted the same ΔT1-T2 variables. These autoregressive estimates were also included in the following models. Second, we estimated the mental health model (Model 2), in which mental health indicators (life satisfaction, positive functioning, anxiety, and depression symptoms) at T1 predicted ΔT1-T2 in job boredom. Third, we estimated the job boredom model (Model 3), in which job boredom at T1 predicted ΔT1-T2 in mental health indicators (life satisfaction, positive functioning, anxiety, and depression symptoms). Finally, we estimated a reciprocal model (Model 4), which included all the previously mentioned estimates, that is, job boredom at T1 predicted the ΔT1-T2 in all four mental health indicators, and all mental health indicators at T1 predicted the ΔT1-T2 in job boredom. We compared the nested models using the Satorra-Bentler χ2 difference test to determine the best-fitting model for our data. In the best-fitting model, we regressed all the outcome variables (ΔT1-T2) to age, gender, education, job transition, and telework to control their effects.

## Results

Table 1 presents the sample characteristics, and Table 2 shows the descriptives and correlations. The LCSM comparisons are shown in Table 3. The Satorra-Bentler χ^2^ difference test suggested that the reciprocal model (Model 4; Table 3) provided a superior fit over the other models. After adding the control variables of age, gender, education, job transition, and telework, the model fit remained acceptable (χ^2^(*df*) = 1519.381 (732), root mean square error of approximation (RMSEA) = 0.046, comparative fit index (CFI) = 0.913, Tucker–Lewis index (TLI) = 0.904, standardized root mean squared residual (SRMR) = 0.076). *n* = 3 participants were excluded from the final model due to the default listwise deletion of missing covariate variables.

**Table 1:** Sample characteristics (*n* = 516)

	**Unweighted descriptives**	**Weighted descriptives**
**Mean age**	31	30
**Gender**		
Women	62%	46%
Men	38%	54%
**Education**		
High	73%	66%
Low	27%	34%
**Weekly working hours**	37	37
**Telework**		
Not at all	53%	57%
¼ of working time	10%	8%
½ of working time	7%	6%
¾ of working time	13%	13%
Approximately all the time	17%	16%
**Employment**		
Full-time	86%	84%
Part-time, occasional, or partly laid off	14%	16%
**Sector**		
Public	35%	31%
Private	60%	62%
Non-profit or other	5%	7%
**Job transition**	24%	21%

Education (High = university or doctoral degree, low = primary or secondary education); job transition = changed jobs between T1 and T2

**Table 2:** Means, standard deviations, scales, Cronbach’s alphas, and correlations (*n* = 516)

Variable	Mean	SD	Scale	α	1	2	3	4	5	6	7	8	9	10	11	12	13	14
1. Job boredom T1	3.31	1.43	0–6	0.81	-													
2. Job boredom T2	3.29	1.37	0–6	0.75	0.65	-												
3. Life satisfaction T1	3.96	0.84	1–5	-	-0.29	-0.31	-											
4. Life satisfaction T2	3.94	0.83	1–5	-	-0.25	-0.27	0.67	-										
5. Positive functioning T1	5.52	0.98	1–7	0.92	-0.31	-0.31	0.74	0.59	-									
6. Positive functioning T2	5.56	0.96	1–7	0.93	-0.35	-0.30	0.64	0.77	0.73	-								
7. Anxiety symptoms T1	0.73	0.62	0–3	0.88	0.29	0.33	-0.50	-0.45	-0.44	-0.41	-							
8. Anxiety symptoms T2	0.69	0.63	0–3	0.89	0.32	0.29	-0.44	-0.56	-0.43	-0.56	0.54	-						
9. Depression symptoms T1	1.29	0.63	1–5	0.94	0.17	0.24	-0.57	-0.45	-0.60	-0.40	0.40	0.39	-					
10. Depression symptoms T2	1.26	0.60	1–5	0.94	0.22	0.23	-0.52	-0.63	-0.42	-0.59	0.40	0.49	0.48	-				
11. Gender	-	-	0–1	-	-0.01	0.01	-0.09	-0.06	-0.03	-0.04	0.28	0.21	0.06	0.02	-			
12. Age	30.22	4.05		-	0.03	-0.09	0.04	0.02	-0.03	0.01	-0.03	-0.02	-0.10	-0.01	0.03	-		
13. Education	-	-	0–1	-	0.07	0.01	0.10	0.07	0.12	0.06	-0.09	0.01	-0.04	-0.13	0.11	0.16	-	
14. Telework	2.24	1.60		-	0.13	0.15	0.04	0.08	0.06	0.04	0.05	0.02	-0.08	-0.11	-0.01	-0.01	0.28	-
15. Job transition	-	-	0–1	-	0.07	0.04	-0.11	-0.09	-0.02	-0.06	0.13	0.18	0.11	0.16	0.23	-0.20	0.06	0.01

*SD* Standard deviation, *α* Cronbach’s alpha, Correlations |0.12–0.13|. are significant at *p* < 0.05, correlations |0.15–0.18|. are significant at *p* < 0.01, correlations |0.20|. and greater are significant at *p* < 0.001. Due to rounding, the correlation between gender and education is significant at *p* < 0.05, despite the coefficient being 0.11

**Table 3:** Model fit indices and model comparison using Satorra-Bentler χ^2^ difference test (*n* = 516)

Model	χ^2^	df	RMSEA	CFI	TLI	SRMR	Comparison	Δχ^2^ (*p*-value)	Preferred model
M1. Autoregressive^a^	1302.034	585	0.049	0.915	0.908	0.101			
M2. Mental health^b^	1286.184	581	0.048	0.916	0.909	0.097	M1-M2	15.908 (0.003)	M2
M3. Job boredom^c^	1266.264	581	0.048	0.919	0.912	0.086	M1-M3	56.898 (< 0.001)	M3
M4. Reciprocal^d^	1250.804	577	0.048	0.920	0.913	0.083	M4-M1	60.437 (< 0.001)	M4
							M4-M2	54.645 (< 0.001)	M4
							M4-M3	15.405 (0.004)	M4

*χ*^*2*^ chi square fit statistic, *df* degree of freedom, *RMSEA* root mean square error of approximation, *CFI* comparative fit index, *TLI* Tucker–Lewis fit index, *SRMR* standardized root mean square residual, *Δχ*^*2*^ chi square difference

^a^Estimated autoregressive paths (T1 to ΔT1-T2) for job boredom, life satisfaction, positive functioning, anxiety, and depressive symptoms

^b^Estimated autoregressive paths and paths from mental health indicators T1 (life satisfaction, positive functioning, anxiety, and depression symptoms) to ΔT1-T2 in job boredom

^c^Estimated autoregressive paths and paths from job boredom T1 to ΔT1-T2 in mental health indicators (life satisfaction, positive functioning, anxiety, and depression symptoms)

^d^Estimated all previously mentioned paths

**Fig. 1:**
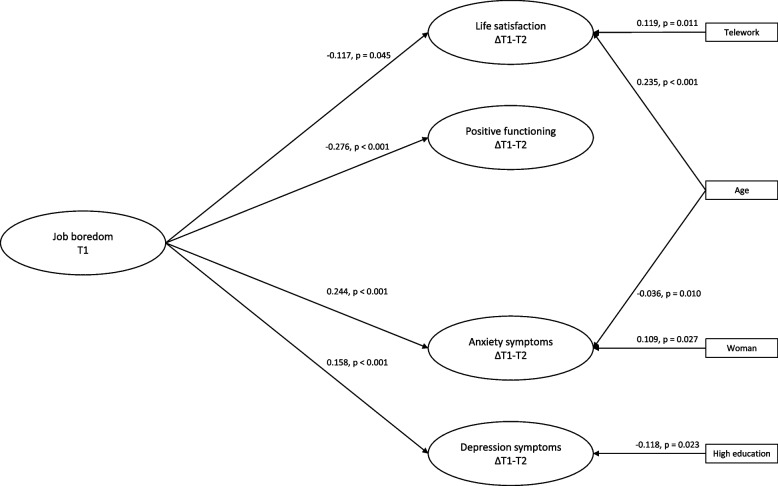
Standardized path estimates of the reciprocal model (Model 4; *n* = 513). Note. For the sake of parsimony, the following estimates were omitted from the figure: 1) the significant autoregressive paths for job boredom, life satisfaction, positive functioning, anxiety, and depression symptoms, 2) the non-significant estimates of T1 life satisfaction, positive functioning, anxiety, and depression symptoms to ΔT1-T2 job boredom, 3) the non-significant estimates of job transition

The statistically significant associations in the final model (Model 4) are illustrated in Fig. 1. All path estimates of this model are presented in Table 4. The estimates in Table 4 show that the more there was job boredom at T1 (i.e., baseline level), the more life satisfaction (*β* = -0.117, *p* = 0.045) and positive functioning (*β* = -0.276, *p* < 0.001) decreased, and the more anxiety (*β* = 0.244, *p* < 0.001) and depression symptoms (*β* = 0.158, *p* < 0.001) increased. Higher age (*β* = 0.235, *p* < 0.001) and teleworking (*β* = 0.119, *p* = 0.011) were associated with increases in life satisfaction. Higher age was also associated with decreases in anxiety symptoms (*β* = -0.036, *p* = 0.010). Being a woman was associated with increases in anxiety symptoms (*β* = 0.109, *p* = 0.027), and having a higher education was associated with decreases in depression symptoms (*β* = -0.118, *p* = 0.023). None of the mental health indicators at T1 were significantly associated with changes in job boredom.

**Table 4:** Standardized estimates and standard errors for cross-lagged estimates in the reciprocal model (Model 4; *n* = 513)

	**β**	**SE**	***p*-value**
Autoregressions			
Job boredom	-0.534	0.057	< 0.001
Life satisfaction	-0.415	0.046	< 0.001
Positive functioning	-0.558	0.070	< 0.001
Anxiety symptoms	-0.616	0.059	< 0.001
Depression symptoms	-0.616	0.130	< 0.001
Cross-lagged paths			
Job boredom → Δ Life satisfaction	-0.117	0.058	0.045
Job boredom → Δ Positive functioning	-0.276	0.071	< 0.001
Job boredom → Δ Anxiety symptoms	0.244	0.058	< 0.001
Job boredom → Δ Depression symptoms	0.158	0.040	< 0.001
Life satisfaction → Δ Job boredom	0.103	0.077	0.183
Positive functioning → Δ Job boredom	-0.117	0.120	0.328
Anxiety symptoms → Δ Job boredom	0.165	0.100	0.099
Depression symptoms → Δ Job boredom	0.131	0.073	0.075

*Δ* = *T1-T2* change in a latent variable, *β* standardized estimate, *SE* standard error

The negative estimates of the autoregressions are caused by latent change score modelling. The interpretation of these estimates is that higher levels of the T1 variable are associated with lower levels of average change in the same variable

Next, we tested whether the path coefficients from job boredom to the different mental health indicators differed statistically significantly by using the model constrain command in Mplus [30]. By this, we tested whether job boredom was more strongly associated with certain mental health indicators than with others. To ensure meaningful comparisons, we reversed the anxiety and depression items so that job boredom was negatively associated with every mental health indicator in the model. Table 5 shows the results of the path comparisons. The path estimate between job boredom at T1 and ΔT1-T2 in life satisfaction was significantly different to ΔT1-T2 in positive functioning (B difference = 0.122, *p* = 0.017) and to ΔT1-T2 in anxiety symptoms (B difference = 0.162, *p* = 0.014) but not to ΔT1-T2 in depression symptoms (B difference = 0.074, *p* = 0.055). These results show that job boredom was more strongly related to changes in positive functioning and anxiety symptoms than to changes in life satisfaction.

**Table 5:** Unstandardized path estimate comparisons in the reciprocal model (Model 4; *n* = 513)

Path estimate comparison of outcomes (from T1 job boredom)	B difference	*p*-value
Δ Life satisfaction vs Δ Positive functioning	0.122	0.017
Δ Life satisfaction vs Δ Anxiety symptoms	0.162	0.014
Δ Life satisfaction vs Δ Depression symptoms	0.074	0.055
Δ Positive functioning vs Δ Anxiety symptoms	0.040	0.647
Δ Positive functioning vs Δ Depression symptoms	-0.048	0.358
Δ Anxiety symptoms vs Δ Depression symptoms	-0.087	0.173

## Discussion

This study contributes to the understanding of the temporal relationships between job boredom and four mental health indicators: life satisfaction, positive functioning, anxiety, and depression symptoms. Our results suggest that boredom at work potentially harms general mental health as it may lead to decreases in life satisfaction and positive functioning and increases in anxiety and depression symptoms.

Drawing from Hobfoll’s [24] COR theory, job boredom, as a low-energy state, may lead employees to invest less in work activities that would promote their well-being [34]. As such, job boredom may prevent future resource gains and make employees vulnerable to future losses, which in our study was indicated by the subsequent decline in general mental health indicators. As we approached mental health as a holistic concept, our findings provide more nuanced evidence of the potential impact of job boredom on mental health. Job boredom may potentially have stronger associations with activation-based states of positive functioning and anxiety symptoms than with life satisfaction (Table 5) [35]. This may be because one of the main drivers of job boredom is a lack of stimulation from the environment [36], and as job boredom represents a lack of energy, it may particularly impact states that are high in arousal. Whereas previous cross-sectional studies have shown, for instance, that job boredom and depression symptoms correlate within-time [18, 19, 23], our findings reveal more about the potential temporal associations between job boredom and mental health indicators. Our results indicate job boredom may be more likely to deteriorate general mental health, than vice versa. Specifically, our findings reveal that job boredom may not only increase depression, but it may also increase anxiety and decrease positive mental health: life satisfaction and positive functioning. Our study brings a significant contribution by showing that mitigating job boredom is not only a way to mitigate negative mental health symptoms, as previous studies suggest, but also a potential way to promote positive mental health, even in the absence of mental illness symptoms. Furthermore, we show how the effects may not be the same across negative and positive mental health indicators, which highlights the importance of studying mental health as a holistic concept.

Future research would benefit from examining further the potential reciprocal effects between job boredom and mental health. While we found statistically significant path estimates only from job boredom to changes in mental health indicators, and not from mental health indicators to changes in job boredom, the reciprocal model still provided the best fit for our data (Table 3). This indicates a potential presence of (some) associations also from general mental health to job boredom, albeit in this study all such associations were below the common threshold of statistical significance (*p* < 0.05). Therefore, our results do not exclude the possibility of general mental health also spilling over into job boredom, thus necessitating future research on the topic.

Our study is not without limitations. First, while we examined associations over time across two-time points, future studies could provide more information regarding the potential direction of causal relationships by conducting longitudinal studies with more waves [37]. Yet, our study is one of the few that has separated job boredom and it’s potential mental health outcomes across time. Also, the inherent issue in survey research is the potential biasing effect of an omitted variable that could explain the found associations between prior levels and subsequent changes in the examined variables. For example, burnout may predict both job boredom [27] and lower mental health [6]. However, we controlled for various demographic (age, gender, and education) and job-related variables, such as telework and job transition to combat the potential effects of the omitted variable bias. Third, we measured life satisfaction with only one item, and thus the results regarding life satisfaction may have been more influenced by potential measurement error, whereas for the other measures, we took the effect of measurement error into account by modelling latent variable’s with several survey items. However, the single-item life satisfaction measure that we used has been found to perform very similarly to the multiple-item satisfaction with life scale [38]. Additionally, our study takes place during the later stages of the COVID-19 pandemic in Finland. This context may have led to more decreases in well-being as the pandemic was generally challenging for workers' well-being [3]. Yet, given that we did not focus on mean changes but rather on the associations between predictors and changes in the outcome variables, we do not expect this aspect to affect our findings. Furthermore, job boredom has previously been associated with mental health indicators such as depression [23] and we controlled the effects of teleworking to account for some of the influence of the COVID-19 pandemic. Lastly, our study focused on participants who were mostly highly educated, in full-time employment, and between the ages of 23 to 34 at baseline. Thus, our results are best generalized in similar groups. Previous studies [1, 3] have shown job boredom to be more prevalent among younger workers, but job boredom studies overall would benefit from looking at different age groups as well as different occupational groups to deepen the understanding of the relationship between job boredom and mental health indicators.

Despite these limitations, our findings provide novel insights regarding the associations between job boredom and mental health indicators. Notably, job boredom may deteriorate mental health. Despite occasional boredom at work being generally harmless [2], the harm caused by working environments that are persistently low in activation should not be underestimated. While increased stress and workload are typically associated with burnout, lack of challenges and activation may also result in job boredom and adverse mental health effects. From the COR perspective, job crafting – proactively customizing one’s job by making changes to one’s tasks and interactions with others at work [39] – is one potential practical approach to mitigating job boredom [19, 34]. Young adults have high expectations of developing their careers [40] and organizations should negotiate and provide job content that provides inspiring and meaningful tasks.

## Conclusions

Overall, our findings contribute to the job boredom literature by providing evidence regarding inferences about the temporal associations between job boredom and mental health. Our study indicates that job boredom leads to decreased general mental health by reducing life satisfaction and positive functioning as well as increasing anxiety and depression symptoms. While our study suggests that job boredom may lead to decreased general mental health, the potential effects of general mental health on job boredom should be studied further.

### Supplementary Information

**Supplementary Material 1. **

## Data Availability

The data will be made available through Finnish Social Science Data Archive after 2025.
