# Supplementary material for: Job boredom as an antecedent of four states of mental health: life satisfaction, positive functioning, anxiety, and depression symptoms among young employees – a latent change score approach
Source: BMC Public Health. 2024 Mar 27;24:907. doi: 10.1186/s12889-024-18430-z (PMC10967055; doi:10.1186/s12889-024-18430-z)
Supplement: Supplementary file 1 — Supplementary Material 1. [file 12889_2024_18430_MOESM1_ESM.docx]

**1. Assessment of non-respondents**

The population structure retrieved from Statistics Finland in December 2020 is presented in the table below. Age, gender, and residential area are shown in the columns from T1 non-respondents (*N* = 10 206), all T1 respondents (*N* = 1794), and the study sample (*N* = 516).

|  | Population structure | T1 non-respondents  (*N* = 10 206) | T1 respondents  (*N* = 1794) | Study sample  (*N* = 516) |
| --- | --- | --- | --- | --- |
| Age:  23-25  26-28  29-31  32-34 | 23%  26%  26%  25% | 19%  26%  26%  29% | 17%  24%  28%  31% | 12%  27%  29%  32% |
| Gender:  Men  Women | 49%  51% | 55%  45% | 39%  61% | 38%  62% |
| Area:  South  East  West  North | 56%  10%  22%  12% | 53%  10%  25%  12% | 58%  9%  23%  10% | 59%  8%  24%  9% |

Those who responded at T1 were slightly older (*M* = 29.38, *SD* = 3.37) compared to those who did not respond (*M* = 29.11, *SD* = 3.40). While this age difference was significant (*t* = -3.040, *df* = 1198, *p* = 0.002), the difference is relatively small. Also, men are slightly underrepresented in T1 respondents.

To further assess the non-response bias in our study, we examined the differences regarding our study variables between employees who responded at T1 (*N* = 801) and employees who responded also at the follow-up, at T2 (*N* = 533). Unemployed were left out of the comparison analysis as they were not instructed not to answer the work-related items in the survey. Those who responded to the follow-up reported slightly higher levels of positive functioning (*M* = 5.57, *SD* = 0.95) compared to employees who only responded to T1 (*M* = 5.42, *SD* = 0.99). The difference is significant (*t* = -2.583, *df* = 1331, *p* = 0.010) but relatively small considering that positive functioning was measured on a 7-point scale. There were no statistically significant mean differences in anxiety symptoms between those who responded to the follow-up (*M* = 0.73, *SD* = 0.62) compared to T1 respondents (*M* = 0.76, *SD* = 0.68), *t* = 0.686, *df* = 1331, *p* = 0.493; in depression symptoms (*M* = 1.24, *SD* = 0.52) compared to T1 respondents (*M* = 1.28, *SD* = 0.59), *t* = 1.151, *df* = 1331, *p* = 0.250; in life satisfaction (*M* = 4.00, *SD* = 0.82) compared to T1 respondents (*M* = 3.93, *SD* = 0.82), *t* = -1.453, *df* = 1329, *p* = 0.146; and in job boredom (*M* = 3.37, *SD* = 1.42) compared to T1 respondents (*M* = 3.28, *SD* = 1.44), *t* = -1.121, *df* = 1329, *p* = 0.262.

Also, there were no statistically significant differences in mean ages between those who responded to follow-up (*M* = 29.53, *SD* = 3.19) compared to T1 respondents (*M* = 29.45, *SD* = 3.45), *t* = -0.439, *df* = 1200.308, *p* = 0.661; and weekly working hours (*M* = 36.48, *SD* = 7.37) compared to T1 respondents (*M* = 36.43, *SD* = 7.99), *t* = -0.116, *df* = 1322, *p* = 0.907). Men were slightly more underrepresented in follow-up (38% men; 62% women) as compared to T1 respondents (45% men; 55% women).

Overall, differences between respondents and non-respondents were small and we do not expect them to bias our findings. Furthermore, differences in gender and age were addressed by weighting responses in the analyses to better correspond with the Finnish population structure. Residential areas were deemed to be representative of respondents.

**2. Measures**

| **Job boredom** (DUBS; Reijseger et al., 2013)  **Scale:** 0 = Never, 1 = few times in a year, 3 = once a month, 4 = few times in a month,  5 = once a week, 6 = daily |
| --- |
| At work, time goes by very slowly |
| I feel bored at my job |
| During work time I daydream |

| **Positive functioning** (The Flourishing Scale; Diener et al., 2010)  **Scale:** 1 = Fully disagree, 2 = Disagree, 3 = Slightly disagree, 4 = Neither agree nor disagree, 5 = Slightly agree, 6 = Agree, 7 = Fully agree |
| --- |
| I lead a purposeful and meaningful life |
| My social relationships are supportive and rewarding |
| I am engaged and interested in my daily activities |
| I actively contribute to the happiness and well-being of others |
| I am competent and capable in the activities that are important to me |
| I am a good person and live a good life |
| I am optimistic about my future |
| People respect me |

| **Anxiety symptoms** (GAD-7; Spitzer et al., 2006)  **Scale:** 0 = Not at all, 1 = Several days, 2 = More than half the days, 3 = Nearly every day |
| --- |
| Feeling nervous, anxious or on edge |
| Not being able to stop or control worrying |
| Worrying too much about different things |
| Trouble relaxing |
| Being so restless that it is hard to sit still |
| Becoming easily annoyed or irritable |
| Feeling afraid as if something awful might happen |

| **Life satisfaction**  **Scale:** 1 = Strongly dissatisfied, 2 = dissatisfied, 3 = neither satisfied nor dissatisfied,  4 = Satisfied, 5 = Strongly satisfied |
| --- |
| Overall, how satisfied are you with your life? |

| **Depression symptoms** (4DSQ; Terluin et al., 2006)  **Scale:** 1 = No, 2 = Sometimes, 3 = Regularly, 4 = Often, 5 = Very often or constantly  **Instruction:** During the past week, did you feel… |
| --- |
| …that everything is meaningless |
| …that life is not worth while |
| …that you would be better off if you were dead |
| …that you can't enjoy anything anymore |
| …that there is no escape from your situation |
| …did you ever think "If only I was dead" |

**References:**

Diener, E., Wirtz, D., Tov, W., Kim-Prieto, C., Choi, D. W., Oishi, S., & Biswas-Diener, R. (2010). New well-being measures: Short scales to assess flourishing and positive and negative feelings. Social indicators research, 97(2), 143–156. doi:https://doi.org/10.1007/s11205-009-9493-y

Reijseger, G., Schaufeli, W. B., Peeters, M. C., Taris, T. W., van Beek, I., & Ouweneel, E. (2013). Watching the paint dry at work: psychometric examination of the Dutch Boredom Scale. Anxiety Stress Coping, 26(5), 508–525. doi:10.1080/10615806.2012.720676

Spitzer, R. L., Kroenke, K., & Williams, J. B. (2006). A brief measure for assessing generalized anxiety disorder: the GAD-7. Archives of internal medicine, 166(10), 1092–1097. doi:doi:10.1001/archinte.166.10.1092

Terluin, B., van Marwijk, H. W., Ader, H. J., de Vet, H. C., Penninx, B. W., Hermens, M. L., . . . Stalman, W. A. (2006). The Four-Dimensional Symptom Questionnaire (4DSQ): a validation study of a multidimensional self-report questionnaire to assess distress, depression, anxiety and somatization. BMC Psychiatry, 6, 34. doi:10.1186/1471-244X-6-34

**3. Parcelling process for anxiety and depression measures.**

We followed the recommendations of Little et al. (2013) for subset-item-parcelling. The unidimensionality of both (anxiety and depression) measures has been established previously (anxiety: Spitzer et al., 2006; depression: Terluin et al., 2006) and to avoid estimation bias, we constructed three parcels to indicate a latent structure (Matsunaga, 2008). We used the balancing approach for assigning items to the parcels. The item with the highest loading was paired with the item with the lowest in order to create equivalent replicates of the overall factor structure. We deemed this approach appropriate because the measures were unidimensional (Little et al., 2013).

**Supplementary table 1**. Item loadings and assigned parcels for anxiety measure (*n* = 516).

| Item | Loading | Parcel |
| --- | --- | --- |
| 1 | 0.716 | 1 |
| 3 | 0.692 | 2 |
| 2 | 0.682 | 3 |
| 4 | 0.662 | 3 |
| 6 | 0.531 | 3 |
| 5 | 0.459 | 2 |
| 7 | 0.333 | 1 |

Note. Model fit: χ2 = 231.828 (*df* = 81), RMSEA = 0.060, CFI = 0.918, TLI = 0.908, SRMR = 0.062

**Supplementary table 2.** Item loadings and assigned parcels for depression measure (*n* = 516).

| Item | Loading | Parcel |
| --- | --- | --- |
| 2 | 0.674 | 1 |
| 1 | 0.668 | 2 |
| 3 | 0.604 | 3 |
| 5 | 0.583 | 3 |
| 6 | 0.565 | 2 |
| 4 | 0.557 | 1 |

Note. Model fit: χ2 = 221.004 (*df* = 57), RMSEA = 0.075, CFI = 0.910, TLI = 0.896, SRMR = 0.083

**References:**

Little, T. D., Rhemtulla, M., Gibson, K., & Schoemann, A. M. (2013). Why the items versus parcels controversy needn’t be one. Psychological methods, 18(3), 285.

Matsunaga, M. (2008). Item parceling in structural equation modeling: A primer. *Communication methods and measures*, 2(4), 260-293.

Spitzer, R. L., Kroenke, K., & Williams, J. B. (2006). A brief measure for assessing generalized anxiety disorder: the GAD-7. Archives of internal medicine, 166(10), 1092-1097. doi:doi:10.1001/archinte.166.10.1092

Terluin, B., van Marwijk, H. W., Ader, H. J., de Vet, H. C., Penninx, B. W., Hermens, M. L., . . . Stalman, W. A. (2006). The Four-Dimensional Symptom Questionnaire (4DSQ): a validation study of a multidimensional self-report questionnaire to assess distress, depression, anxiety and somatization. BMC Psychiatry, 6, 34. doi:10.1186/1471-244X-6-34

**4. Measurement invariance for latent measures (job boredom, positive functioning, anxiety, and depression symptoms). The constructs for the anxiety and depression measures were parcelled.**

In the configural model, all the estimates are free. In the metric model, item loadings are set to be equal across time. Finally, in the scalar model, item loadings and intercepts are set to be equal across time. The Satorra-Bentler χ^2^ difference test showed that the constrained models (metric and scalar) did not significantly differ from previous models with fever parameter constraints.

|  | **χ^2^** | **df** | **Δχ^2^ (p-value)** | **RMSEA** | **CFI** | **TLI** | **SRMR** |
| --- | --- | --- | --- | --- | --- | --- | --- |
| Configural | 940.910 | 482 | - | 0.043 | 0.940 | 0.931 | 0.047 |
| Metric | 944.096 | 495 | 12.431 (0.493) | 0.042 | 0.942 | 0.934 | 0.049 |
| Scalar | 957.591 | 508 | 13.398 (0.418) | 0.041 | 0.942 | 0.935 | 0.049 |

Note. χ^2^ = chi square fit statistic; df = degree of freedom; RMSEA = root mean square error of approximation; CFI = comparative fit index; TLI = Tucker-Lewis fit index; SRMR = standardized root mean square residual; Δχ^2^ = chi square difference.

Configural model: all estimates are free.

Metric model: item loadings are constrained to be equal between T1 and T2.

Scalar model: item loadings and intercepts are constrained to be equal between T1 and T2.
